# Effect of Elective Cesarean Section on Children's Obesity From Birth to Adolescence: A Systematic Review and Meta-Analysis

**DOI:** 10.3389/fped.2021.793400

**Published:** 2022-01-27

**Authors:** Shanshan Zhang, Xiaoyun Qin, Peixuan Li, Kun Huang

**Affiliations:** ^1^Department of Maternal, Child & Adolescent Health, School of Public Health, Anhui Medical University, Hefei, China; ^2^Key Laboratory of Population Health Across Life Cycle (Anhui Medical University), Ministry of Education of the People's Republic of China, Hefei, China; ^3^National Health Commission (NHC) Key Laboratory of Study on Abnormal Gametes and Reproductive Tract, Hefei, China; ^4^Anhui Provincial Key Laboratory of Population Health and Aristogenics, Anhui Medical University, Hefei, China; ^5^Scientific Research Center in Preventive Medicine, School of Public Health, Anhui Medical University, Hefei, China

**Keywords:** cesarean section, obesity, children, systematic review, meta-analysis

## Abstract

**Background:**

Elective cesarean section (ECS) is the most common reason for the increasing cesarean section rate worldwide, and it is reported to be related to adverse short-term and long-term outcomes in both mothers and infants. Findings on the association between ECS and overweight and obesity in children are controversial in recent studies. Therefore, we conducted a systematic review and meta-analysis to examine the effect of ECS on offspring's overweight and obesity.

**Methods:**

PubMed, Science Direct, Web of Science, CNKI (China National Knowledge Infrastructure), Wanfang Database (in Chinese), and China Biology Medicine disc databases were searched using different combinations of three groups of keywords: “elective cesarean section,” “overweight/obesity,” and “children.” Nine cohort studies and 11 independent risk estimates were finally identified.

**Results:**

We have observed significant association between ECS and children's obesity, the total pooled risk ratio (RR) being 1.10 (95% CI: 1.01–1.18; *I*^2^ = 32.4%). In subgroup analysis, ECS was found to be associated with the occurrence of obesity in preschoolers (RR = 1.12, 95% CI: 1.02–1.22; *I*^2^ = 16.8%). Furthermore, it revealed that ECS was related with the high risk of children's obesity where the rate of ECS exceeded 10%. No significant association was observed between ECS and children's overweight, and the RR was 1.12 (95% CI: 0.94–1.30; *I*^2^ = 55.6%).

**Conclusions:**

Overall, it indicated that children born *via* ECS had an increased risk of later-life obesity. Given the global increase in childhood obesity, our findings would provide evidence-based reference for early life intervention on children's obesity.

**Systematic Review Registration:**

https://www.crd.york.ac.uk/prospero/display_record.php?ID=CRD42021267211, identifier: CRD42021267211.

## Introduction

With the development of perinatal medicine, cesarean section (CS) has become an effective means to save women's and infants' lives when encountering severe conditions. In recent years, the CS rate has been increasing rapidly around the world, and has been reported from 26.4% in the WHO Global Survey (2004–08) to 31.2% in the WHO Multi-Country Survey (2010–11) (1).

CS can be divided into elective CS (ECS) and emergency CS (EMCS). EMCS is performed under an acute situation that threatens the life of both mother and fetus and requires immediate termination of pregnancy. Unlike EMCS, ECS is not only based on medical indications, but also up to the request of mothers and their families. CS on maternal request is the most frequently cited reason for the increasing CS rates (2, 3). WHO has estimated that 6.2 million excess—i.e., not medically indicated—CS are being performed each year, 50% of which are in Brazil and China alone (4).

There is no evidence showing the benefits of CS for women or infants who do not require the surgery. In contrast, CS is reported to be associated with adverse short-term and long-term physical and psychological outcomes in both mothers and infants (5). For instance, CS is related to offspring's atopy and asthma, and may also cause reduced intestinal gut microbiome diversity (6), which may contribute to an individual's obesity in later life (7).

Overweight/obesity is widely recognized as a serious public health problem, as it relates to a range of health problems in children and adults, including cardiovascular disease, diabetes, orthopedic disease, low self-esteem, depression, and social marginalization (8). The prevalence of childhood and adult obesity has increased in the past several decades in both developed and developing countries (9). In 2016, the WHO estimated that 41 million children under five were affected by overweight or obesity worldwide, and half of them were in Asia (10). Early childhood overweight and obesity may persist into middle childhood and adolescence (11).

Previous studies have paid close attention to the relationship between CS and offspring's overweight and obesity, but the findings are contradictive. Mueller et al. found that compared to vaginal delivery (VD), CS was associated with a higher rate of offspring's weight gain over the first year (12). Other studies also showed that CS was associated with an increased risk of children's obesity (13, 14). However, a longitudinal cohort study in Ireland argued no association between modes of delivery and children's obesity at the ages of 2 and 5 (15). More than that, when investigating the association between CS and offspring's overweight/obesity, limited studies have distinguished ECS from non-ECS, and the findings are contradictory as well. Cai et al. found that ECS was significantly associated with the risk of infants' overweight or obesity. Masukume et al. revealed that there was a statistically significant association between ECS and obesity with children aged 24 months, but the relationship was not observed when they are 54 months old (16, 17). A Swedish population-based cohort study found no evidence of an association between non-elective or elective CS and obesity in young adulthood (18).

From what has been mentioned above, the association between ECS and offspring's overweight/obesity is controversial. It is regrettably that there is no systematic review on this topic with a matter of concern. The purpose of this study was thus to summarize the available evidence and systematically examine the association between ECS and offspring's weight development from birth to adolescence.

## Methods

### Literature Search and Inclusion Criteria

We searched for relevant publications in PubMed, Science Direct, Web of Science, CNKI (China National Knowledge Infrastructure), Wanfang Database (in Chinese), and China Biology Medicine disc databases from inception to the end of December 2020. We used different combinations on the three clusters of terms: (1) “cesarean/cesarean delivery/section,” “Planned cesarean/cesarean,” “elective cesarean/cesarean”; (2) “weight,” “fat^*^,” “adiposity,” “obesity,” “BMI,” “physical growth,” “physical development”; (3) “child^*^,” “adolescen^*^,” “infant,” “toddler,” “offspring” (Supplemental Table 1). After excluding duplicate records, we scanned titles and abstracts and then retrieved full texts of potentially relevant articles for detailed evaluation. All searches were conducted by Zhang SS and double-checked by Qin XY. The two reviewers reached a consensus at each stage before proceeding.

Studies were considered for inclusion if (1) the study was an original research published in English or in other languages with an English abstract; (2) the study used a cohort (historical or prospective) or case–control design and examined the association between ECS and birth weight or overweight/obesity in offspring; (3) the authors reported mean ± standard deviation of birth weight or RRs/ORs with 95% confidence intervals (CIs) for overweight/obesity in the study.

### Data Extraction and Quality Assessment

Data extraction was independently extracted by two reviewers using a pretested structured form. Discrepancies were resolved on the basis of a consensus discussion between the two reviewers or with the third reviewer (Li PX).

Where possible, we extracted the adjusted effect estimates. If several multivariate-adjusted estimates were available, we only extracted the most adequately adjusted one. If a study longitudinally assessed offspring's overweight or obesity risk based on the same cohort, we extracted all estimates. If a study reported more than one estimate within the same group at different ages, we extracted only the estimate at the longest follow-up period. The extracted information mainly included author, study design, year of publication, country, study sample, children's age, definition of obesity, estimates of outcome, gestational age, and adjustment for confounding factors.

The quality of each included study was independently assessed by two reviewers according to the Newcastle-Ottawa Scale (NOS). The scale awarded a maximum score of 9 to each study: 4 for study group selection, 2 for the comparability between groups, and 3 for the ascertainment of outcome measurement for cohort studies. We defined a score of 7 as high quality, 4–6 as medium, and 3 as low quality. The quality assessment of all included studies is presented in Table 1.

**Table 1:** Characteristics of studies included in the meta-analysis.

**Author**	**Study design**	**Year of publication**	**Country**	**Study sample (n, ECS^**a**^ /VD^**b**^)**	**Children's age**	**ECS^**a**^ rate**	**Definition of obesity**	**Estimates of outcome**	**Gestational age (weeks, ECS^**a**^/VD^**b**^)**	**Adjustment for confounding factors**	**NOS**.
Sitarik et al.	Cohort Study	2020	USA	82/358	10 years	14.3%	BMI ≥ 95th percentile	Obesity: RR (95% CI):1.77 (1.16, 2.72) birth weight (g, ECS/VD): 3,446 ± 758/3,337 ± 529	38.3 ± 1.7/38.9 ± 1.6	Marital status, maternal race, prenatal tobacco smoke exposure, maternal age, maternal BMI, any hypertensive disorders during pregnancy, gestational diabetes, prenatal antibiotic use, child sex, parity, birthweight z-score	8
Maharlouei et al.	Cohort Study	2019	Iran	1,071/1,367	Newborn	/	/	Birth weight (g, ECS/VD): 3,166 ± 442.4/3,213 ± 454.8	38.4 ± 1.2/39.2 ± 1.2	/	8
Masukume et al.	Cohort Study	2019	UK	1,669/12,567	3–5 years	9.2%	IOTF^c^	Obesity: RR(95% CI):0.96 (0.67, 1.38)	/	Maternal age, ethnicity, education, marital status, couple income, infant sex, birth weight, smoking during pregnancy, gestational age, diabetes mellitus, parity, pre-pregnancy BMI	8
Masukume et al.	Cohort Study	2019	New Zealand	618/5,067	4.5 years	9.4%	IOTF^c^	Overweight: RR:(95%CI):1.27 (0.99, 1.63) Obesity: RR (95% CI): 0.85 (0.56, 1.29)	/	Maternal age, education, ethnicity, marital status, infant sex, birth weight, smoking, gestational age, gestational diabetes, parity, pre-pregnancy BMI.	8
Ahlqvist et al.	Cohort Study	2019	Sweden	4,147/89,024	18 years	4%	WHO BMI > 30	Overweight: RR: (95%CI): 0.99 (0.90, 1.08) Obesity: RR (95% CI): 1.02 (0.88, 1.18) birth weight (g, ECS/VD) 3,444 ± 554.7/3,633.1 ± 499.4	38.2 ± 1.3/39.6 ± 1.5	Pre-pregnancy maternal BMI, maternal diabetes at delivery, maternal hypertension at delivery, maternal smoking, parity, parental education, maternal age at delivery, birth weight standardized according to gestational age, preeclampsia, gestational age.	8
Zhou et al.	Cohort Study	2019	China	737/730	4–7 years	50.2%	Central obesity as waist circumference > 75th age- and sex-specific percentile of that for Chinese preschool children	Obesity: OR (95% CI): 1.33 (1.02, 1.72)	/	Maternal age, educational level, BMI in early pregnancy, gestational weight gain, micronutrient supplementation, sex, gestational age, sex-adjusted birthweight-for-gestational age z scores, and age at follow-up visit.	8
Cai et al.	Cohort Study	2018	Singapore	74/505	1 year	10.1%	Risk of overweight: 1sd < BAZ^d^ ≤ 2SD Overweight: BAZ > 2SD	Overweight: OR (95% CI): 2.05 (1.08, 3.90)	/	Ethnicity, maternal age at delivery, maternal educational level, parity, early pregnancy body mass index, antenatal active or passive smoking, hypertensive disorders of pregnancy, gestational diabetes, sex-adjusted birth weight–for–gestational agezscore.	8
Masukume et al.	Cohort Study	2018	Ireland	1,402/6,579	5 years	12.7%	IOTF^c^	Overweight: RR: (95% CI): 1.13 (0.94, 1.35) Obesity: RR (95% CI): 1.30 (0.98, 1.73) birth weight (g, ECS/VD) 3,431 ± 562/3,507 ± 502	38.7 ± 1.7/39.7 ± 1.9	Maternal age, education, ethnicity, marital status, region, infant sex, gestational age, pre-eclampsia, gestational diabetes, parity	8
Black et al.	Cohort Study	2015	UK Scotland	12,355/ 252,917	5 years	3.8%	BMI > the 95th centile	Obesity (RR 95% CI): 1.12 (0.99, 1.26) birth weight (g, ECS/VD) 3,301 ± 494.1/3,379.4 ± 454.6	38.7 ± 1.0/39.8 ± 1.2	Maternal age, gestation at birth, maternal Carstairs decile, maternal smoking status, birth weight, year of delivery, male infant, breastfeeding at 6 weeks, maternal BMI	8

a *ECS, Elective Cesarean Section*.

b *VD, Vaginal Delivery*.

c *IOTF, International Obesity Task Force*.

d *BAZ, BMI-for-age z scores*.

### Statistical Analysis

All analyses were conducted by using stata11. Main outcomes in the current study included offspring's birth weight and later overweight/obesity. When estimating the overall effect of ECS on offspring's overweight/obesity, since the OR values do not define the actual risk of the association of interest, we converted all ORs into RRs by the following formula: $RR=\frac{OR}{\left[\left(1-Pref\right)+\left(pref*OR\right)\right]}$ (RR: risk ratio; OR: odds ratio; Pref: Prevalence of the outcome in the reference group) (19).

We also performed two subgroup analyses to examine the effect of ECS on children's obesity. First, we re-run the analysis stratified by children's age. In age stratification, “preschool-age period” referred to 3–7 years and “adolescence” referred to 10–18 years. Second, we examined the effect of ECS on children's obesity, respectively, in the studies with “high” ECS rate (>10%) and “low” ECS rate (10%).

Forest plot was used to present the RR value of overweight/obesity. We used *I*^2^ to evaluate the statistical heterogeneity between studies. If *I*^2^ < 50%, and the heterogeneity was low and acceptable, then the fixed effect model was used for meta-analysis; if *I*^2^ > 50% and heterogeneity between studies was high, the random effect model was adopted. Publication bias was detected by the funnel plot and Egger's/Begg's test. Sensitivity analysis was introduced to assess the effect of each individual study on the overall estimate. We also used sensitivity analysis to investigate the stability of the outcome. The pooled risk ratios were observed after removing each study. If the pooled risk ratios exceeded the original confidence interval, the finding was regarded as unstable; otherwise, the finding was considered to be stable.

### Protocol Registration

The protocol for this systematic review and meta-analysis has been registered in the PROSPERO (Protocol No. CRD42021267211).

## Results

### Characteristics of the Selected Studies

**Figure 1:**
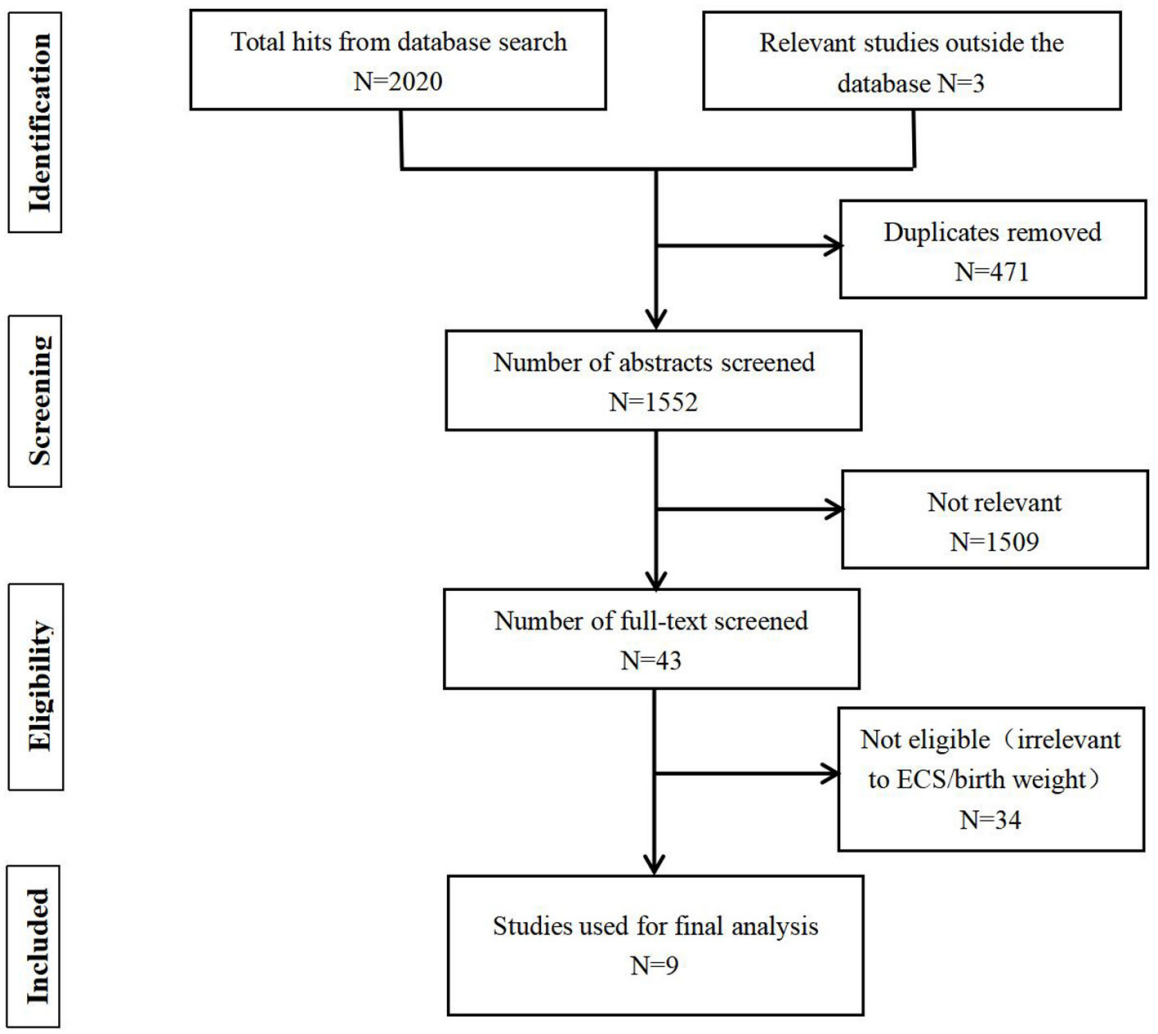
Flowchart on literature search and study selection.

A total of 2,023 studies were obtained through literature search. After deleting the repeated literature, there were 1,552 articles left. After reviewing the titles and abstracts, 43 relevant studies were obtained. After reading the full text, a total of 9 studies met the inclusion criteria and were used for final meta-analysis (16–18, 20–25) (Figure 1).

The characteristics of the nine eligible studies are listed in Table 1. These nine studies were conducted in eight countries and were all cohort studies. With the exception of one study that only covered boys, the others included both boys and girls in their samples. The total sample size was 391,269, ranging between 74 and 252,917 in individual studies. The participants were followed up from birth to median 18 years of age.

### The Association Between ECS and Infants' Birth Weight

**Figure 2:**
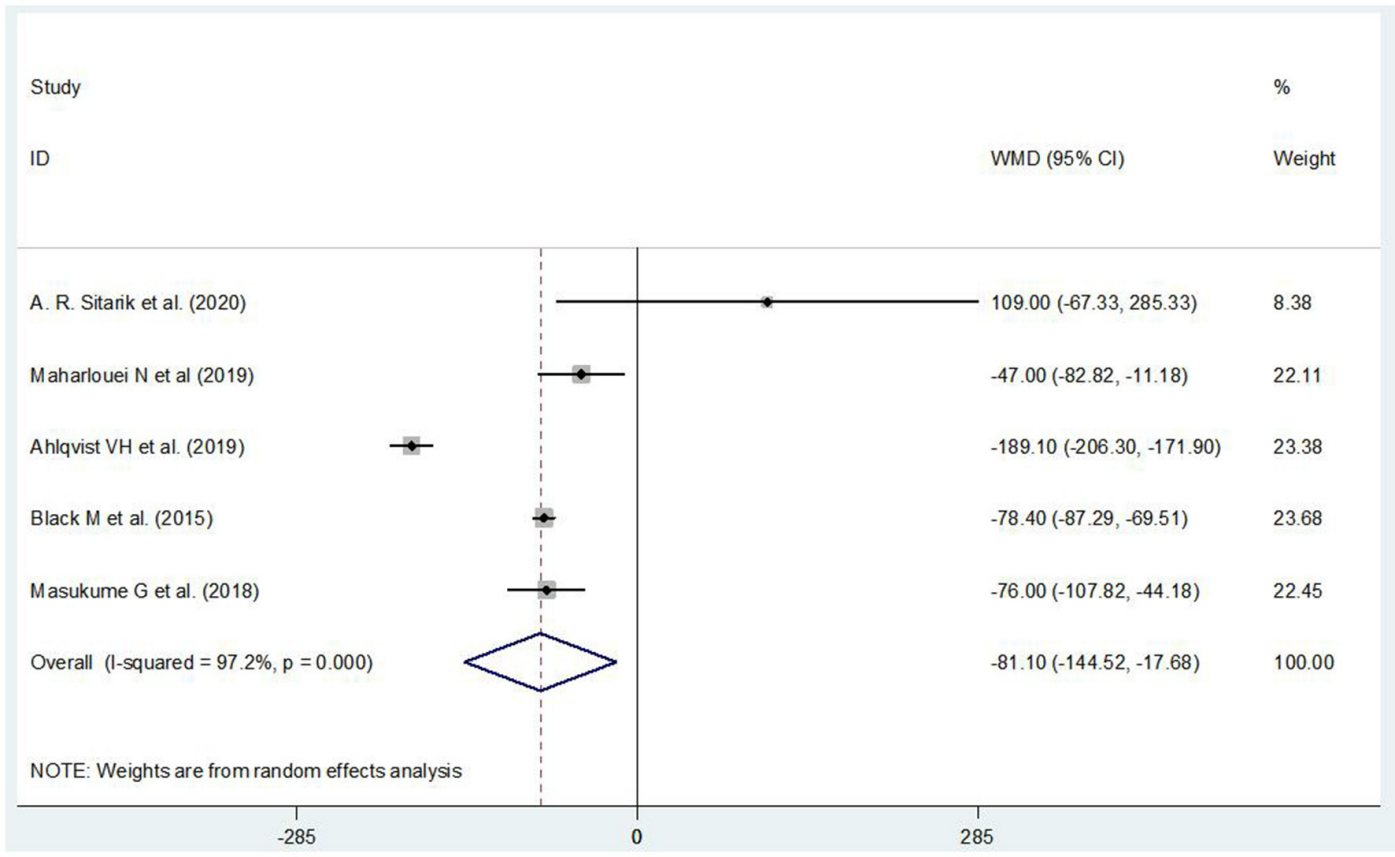
The association between ECS and infants' birth weight.

Five of the 9 cohort studies reported children's birth weight (*n* = 369,282) born *via* ECS and VD. Birth weight in children born *via* ECS was found to be lower than that in children born *via* VD (WMD = −81.10, 95% CI: −144.52, −17.68) (Figure 2). Publication bias was detected by the funnel plot and Egger's test (*P* > |*t*| = 0.986), and it indicated that there was no publication bias. The sensitivity analysis showed that the pooled RRs did not exceed the original confidence interval after removing each study in turn, and indicated that the result was stable.

### The Association Between ECS and Children's Overweight

**Figure 3:**
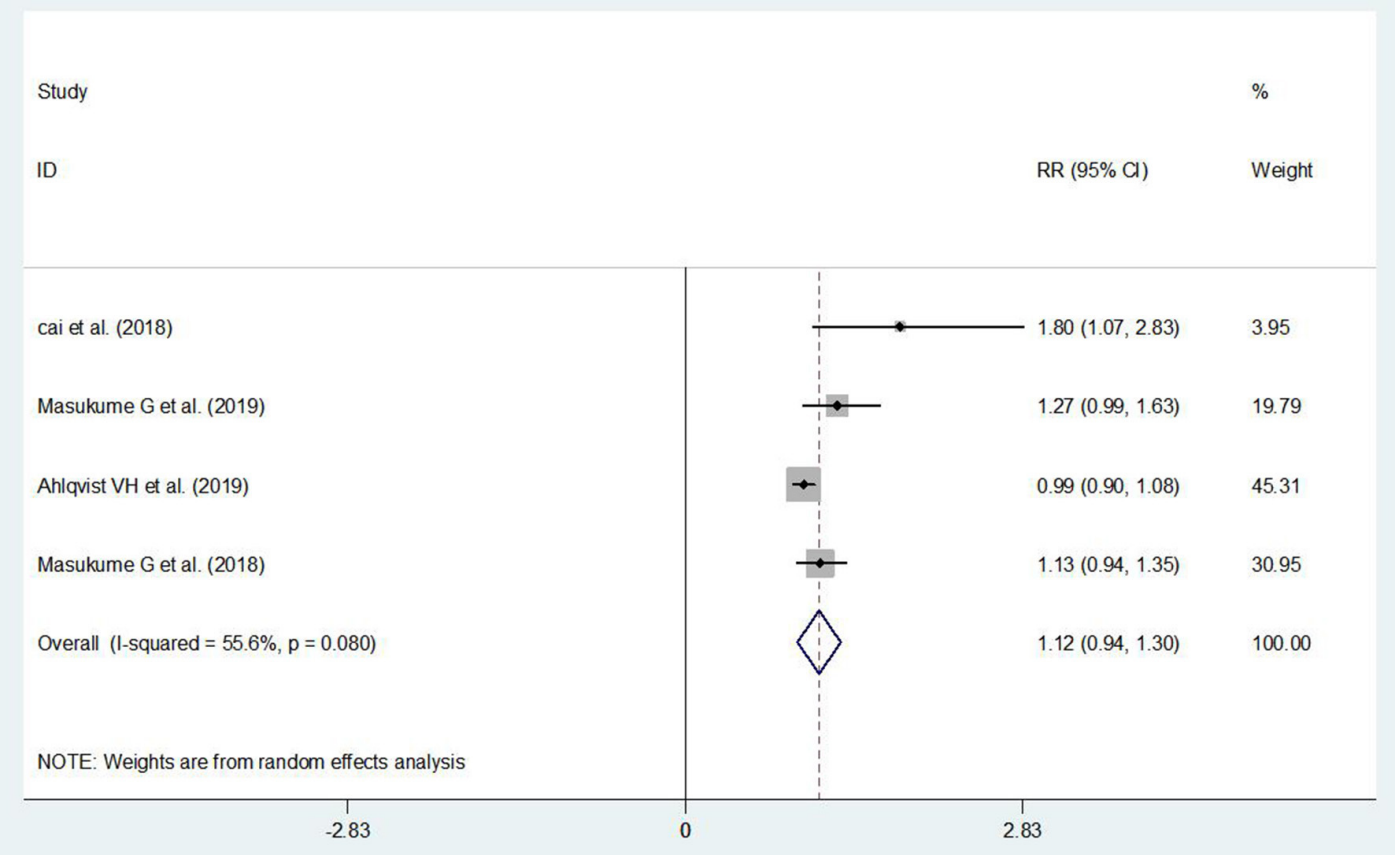
The association between ECS and children's overweight.

It did not show a significant association between ECS and children's overweight (RR = 1.12, 95% CI: 0.94–1.30) (Figure 3). Publication bias was detected by the funnel plot and Egger's test (*P* > |*t*| = 0.001), and it indicated that there was publication bias. Sensitivity analysis had shown that the finding was stable.

### The Association Between ECS and Children's Obesity

**Figure 4:**
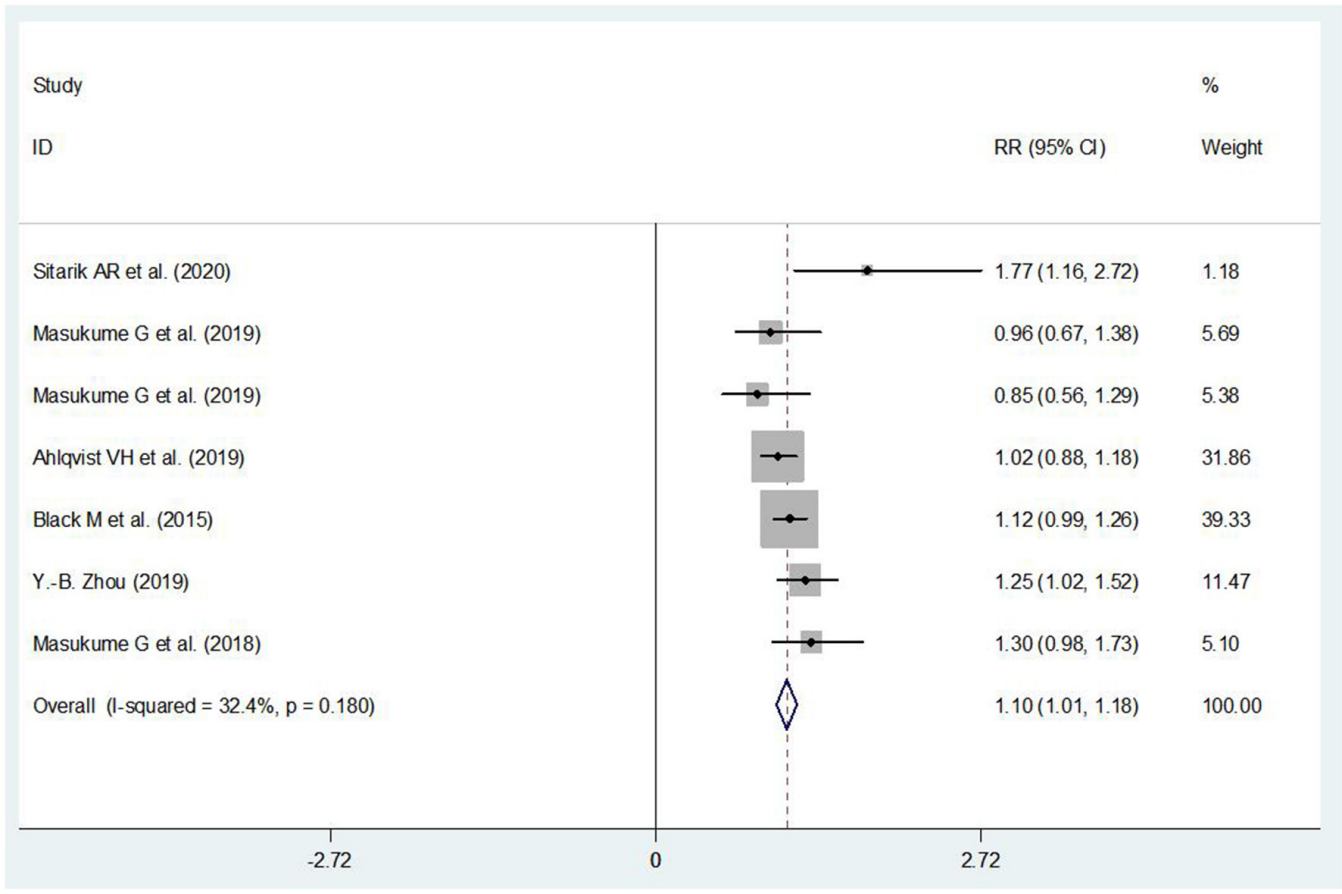
The association between ECS and children's obesity.

Compared with those being born *via* VD, it showed an increased risk of being obesity in children born *via* ECS (RR = 1.10, 95% CI: 1.01, 1.18) (Figure 4). Publication bias was detected by the funnel plot and Egger' s test (*P* > |*t*| = 0.638), and it indicated that there was no statistically significant publication bias, as it was not likely to substantially affect the results.

**Figure 5:**
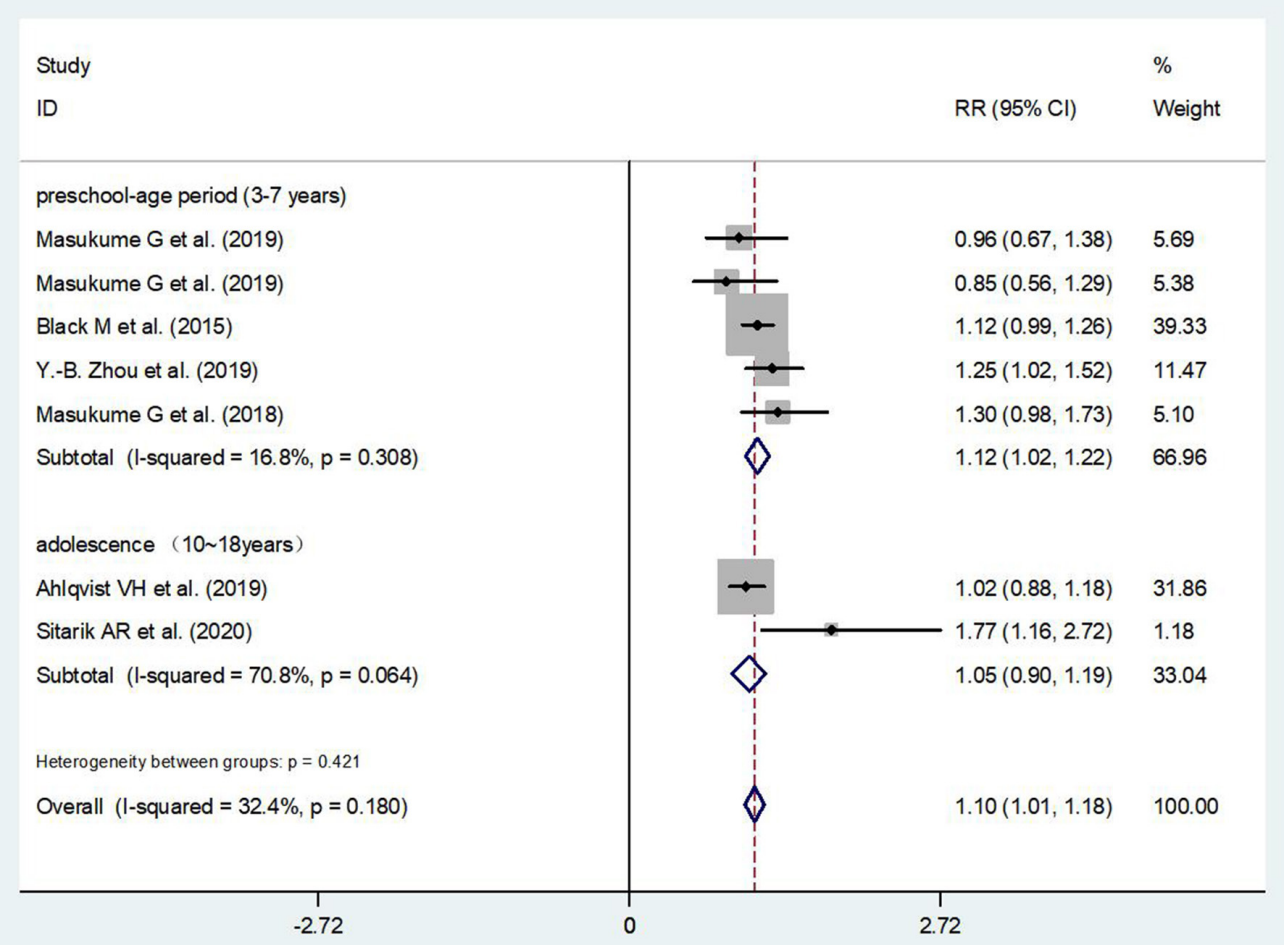
Subgroup analysis on the association between ECS and children's obesity stratified by age.

**Figure 6:**
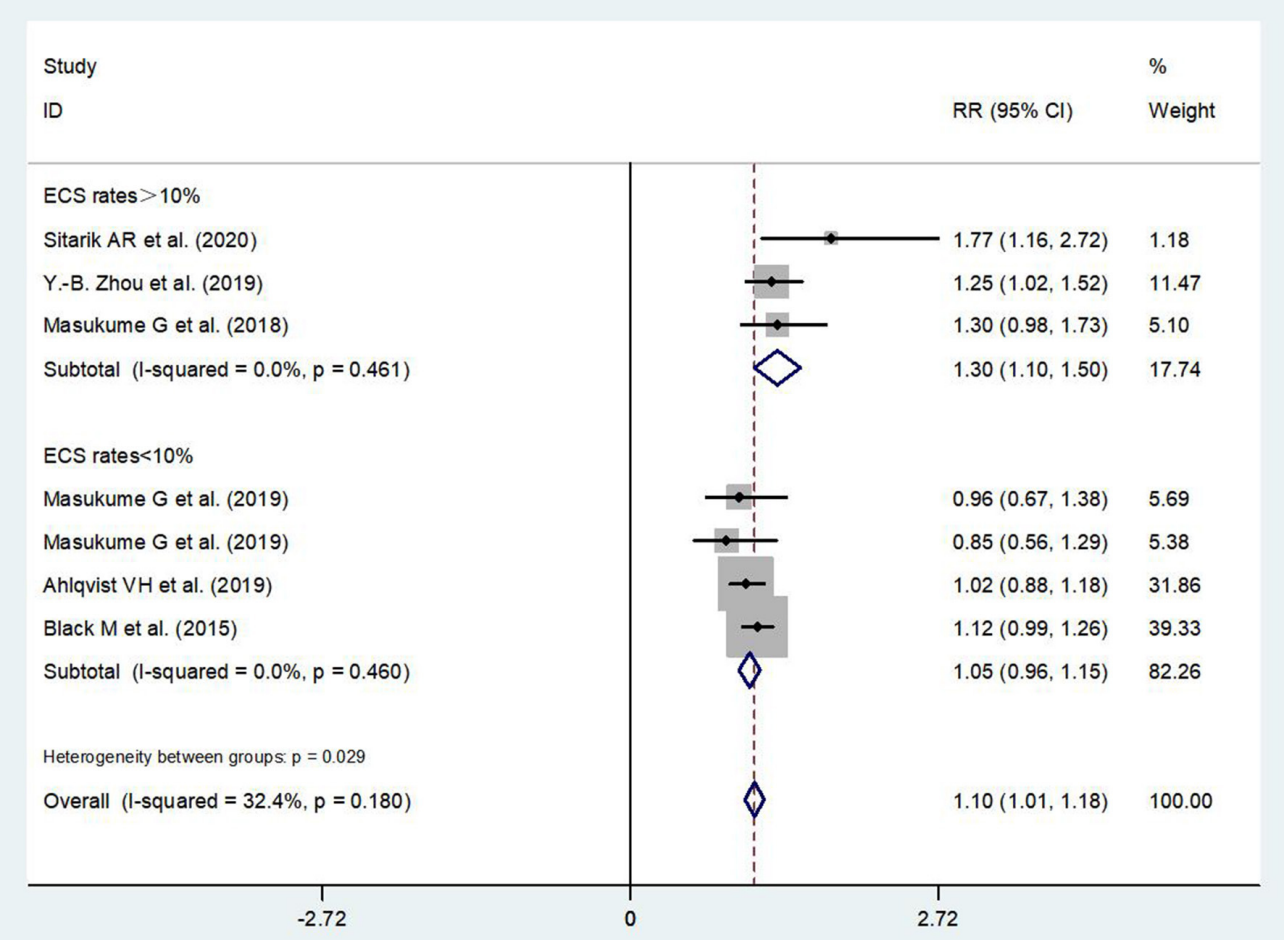
Subgroup analysis on the association between ECS and children's obesity stratified by ECS rate.

According to the available data, we have performed a subgroup analysis on the association between ECS and children's obesity stratified by age. It showed that ECS was associated with a high risk of obesity in children at preschool-age period (RR = 1.12, 95% CI: 1.02–1.22) (Figure 5). In other subgroup analysis, the effect of ECS on children's obesity was found in places where the ECS rate exceeded 10% (RR = 1.30, 95% CI: 1.10–1.50) (Figure 6).

## Discussion

The current study indicated that, compared with children born *via* VD, children delivered by ECS had lower birth weight and an increased risk of obesity from infancy to adolescence. As far as we know, this is the first systematic review and meta-analysis that focuses on the association between ECS and children's long-term weight development.

Our findings on lower birth weight in children born *via* ECS were contrary to the study of Arikan et al. (26), which indicated that the weight of newborns delivered by ECS (ECS on maternal request due to fear of childbirth) is heavier than those born *via* VD. There may exist a bidirectional relationship between ECS and infants' birth weight. On one hand, birth weight might be a result of ECS. If there are serious pregnancy complications that require early termination of pregnancy, the gestational age might be small and thus the birth weight might be accordingly low (2). Although the American Congress of Obstetricians and Gynecologists recommend that that ECS should not be performed before a gestational age of 39 weeks (27), still there are many ECSs conducted before 39 gestational weeks, even for ECS on maternal request (28). On the other hand, birth weight might be the cause of ECS. For instance, if fetal ultrasound indicates the possibility of macrosomia and causes difficulties to have VD, ECS would be advised or requested (2). In the current study, we found that the gestational age of newborns born *via* ECS was about 1 week younger than that of newborns born *via* VD (WMD: −0.93, 95% CI: −1.13, −0.73), but we could not tell the exact causality between the two variables. However, whether birth weight is regarded as a confounder or a mediator, the studies we included had all controlled birth weight in data analysis.

Still, lower birth weight and younger gestational age of newborns delivered by ECS were not enough to indicate children's exact intrauterine development, but the possibility of potential IUGR in children delivered by ECS could not be fully ruled out. It is speculated that the risk of obesity in children born of ECS might be due to the catch-up growth of IUGR infants *via* this operative delivery mode.

The 10% increased risk of obesity identified following ECS compared with VD is consistent with the other studies. Li et al. (29) showed that the overall pooled odds ratio (OR) of overweight/obesity in offspring delivered by CS was 1.33 (95% CI: 1.19, 1.48) compared with those born *via* VD. Although Kuhle et al. (30) did not distinguish CS from ECS and non-ECS, they had performed a meta-analysis in 2015 and found that children born *via* CS had a 34% higher risk of obesity than those born *via* VD.

Recent studies have suggested that the link between delivery modes and offspring's overweight/obesity may be caused by different mother-to-child microbiome at birth. Different exposures to the vaginal, perineal, and fecal microflora in CS (especially ECS) and VD infants are thought to determine the different initial composition intestinal microflora (31). A Norwegian Birth Cohort study examined the relationship between early gut microbiota and children's BMI at age 12 and found that composition of the gut microbiota at days 10 and 730 was significantly associated with childhood BMI (32). Studies have shown that *Escherichia-Shigella* and *Bacteroides* species were underrepresented in infants born by CS (33, 34). A lack of exposure to these microbes at birth may increase the risk of obesity later in life, as biological dysregulation or depletion of the microbes may impede normal energy acquisition and alter metabolic procedures (33–36), whereas in emergency CS, membranes usually have ruptured, with the consequent infants' exposure to vaginal microbiota, as this may partially reduce the risk of later life obesity compared with infants born *via* ECS with intact membranes (16).

Stress hormone triggered by different delivery modes may also play a role. Normal increase in the stress hormone, cortisol, during labor is beneficial for the newborn's adaptation to the extra-uterine environment (37). Research suggests that the lowest stress response is found in infants born *via* ECS, a lower response is found in those born *via* emergency CS, and a high response is found in those born *via* normal VD (38, 39). Studies propose that lower levels of fetal cortisol in ECS may cause metabolic perturbations at birth and future obesity (40). Kiriakopoulos' study found a higher level of growth hormone (GH) in the umbilical cord blood in infants with ECS compared to those with VD. Experimental studies concur that increased GH level is associated with dysfunction of the adipose tissue and thus leads to increased body weight (41).

The subgroup analysis indicated an increased risk of obesity in preschoolers born *via* ECS. This is consistent with the trajectory of body fat development, which continues to grow in childhood and tends to be stable in adolescence (42, 43). Meanwhile, low gut microbiome diversity and richness in infant caused by ECS has widely been reported and lead to the subsequent expansion of adiposity tissue in infancy (33–36). A large amount of evidence supports that obesity during birth and infancy has a great influence on obesity in later life stage, including preschool-age and adolescence (44). Zhou's study had focused on the dynamic changes in children's growth and development and confirmed that CS significantly increased the risk of “progressive obesity” trajectory (OR = 2.50, 95% CI: 1.42, 4.41) at the ages of 10–15 years (45). Due to the limited data, we could not provide powerful findings on the effect of ECS on the occurrence of obesity in adolescents in the current study. More research data are needed to identify the association between ECS and adolescents' obesity.

In our study, the effect of ECS on children's obesity was observed in places where the ECS rate exceeded 10%. Previous studies showed that CS rates at the population level exceeding 10% were not associated with reductions in maternal and newborn mortality any more (46, 47). Our findings toward children's obesity here also provide new evidence on the adverse health outcome caused by cesarean section from the perspective of offspring's growth and development.

There are some strengths in our study. First of all, it is the first systematic analysis on the association between ECS and offspring's long-term weight development. ECS and non-ECS have been clearly distinguished in the current study. The finding is of high public health value without doubt as ECS (especially ECS on maternal request) is a potentially modifiable risk factor for children's obesity. Then, all the included studies are cohort designed, with high quality, and the sample size is large. It allows more opportunities to clarify causal relationship between ECS and children's overweight and obesity. Furthermore, heterogeneity was low in all primary analyses given all studies were observational.

Meanwhile, some limitations must be acknowledged. Because a limited number of studies distinguished ECS from non-ECS when examining the effect of cesarean section on children's obesity, there were a small number of studies included in the current meta-analyses. Thus, the results should be viewed with caution. However, the original studies included were all cohort studies with large sample size covering a wide range of areas; our findings, therefore, would provide credible evidence to a certain extent. Secondly, due to limited information in recruited articles, we were not able to distinguish ECS with medical indications from that without any indications. The former is a necessary surgical procedure to save mothers' and infants' lives, while the latter may violate the eugenics principles and adversely affect the offspring's development (2, 6). The public health significance between the two kinds of ECS, therefore, is absolutely different. Thirdly, the effects of early life exposure on child development are usually sex-specific (48). Few studies had distinguished the children's sex when examining the association between ECS and children's overweight and obesity, which hinders us in exploring the sex-specific effect of ECS on children's weight development. Although all the included studies in the current study had adjusted for children' sex, it was not possible to examine the specific effect of ECS on obesity, respectively, in boys and girls. Moreover, genetic determinants, as well as paternal characteristics, were not fully considered or adjusted in the recruited studies. These are also proven to be potential factors affecting children's overweight/obesity occurrence (49).

In summary, ECS is observed to be related with an increased risk of children's obesity. Under the condition of global high ECS rate, our findings will provide evidence for both obstetricians and women in their informed choice of cesarean section.

## Data Availability Statement

The original contributions presented in the study are included in the article/Supplementary Material, further inquiries can be directed to the corresponding author/s.

## Author Contributions

SZ collated and analyzed the data and was the main author of the first draft. XQ participated in literature search and data analysis. PL participated in literature retrieval and review. KH reviewed and revised the first draft and supervised and led this study. All authors contributed to the article and approved the submitted version.

## Funding

Funding was provided by the National Natural Science Foundation of China (81872630), the University Synergy Innovation Program of Anhui Province (GXXT-2020-067), the Sci-tech Basic Resources Research Program of China (2017FY101107), the Special Project Reproductive Health, Prevention and Control of Major Birth Defects of National Key Research and Development Program (2016YFC1000204-2), the Non-profit Central Research Institute Fund of Chinese Academy of Medical Sciences (2019PT310002), and the Research Fund of Anhui Institute of Translational Medicine (ZHYX2020A001).

## Conflict of Interest

The authors declare that the research was conducted in the absence of any commercial or financial relationships that could be construed as a potential conflict of interest.

## Publisher's Note

All claims expressed in this article are solely those of the authors and do not necessarily represent those of their affiliated organizations, or those of the publisher, the editors and the reviewers. Any product that may be evaluated in this article, or claim that may be made by its manufacturer, is not guaranteed or endorsed by the publisher.
