# Supplementary material for: Effect of Elective Cesarean Section on Children's Obesity From Birth to Adolescence: A Systematic Review and Meta-Analysis
Source: Front Pediatr. 2022 Jan 27;9:793400. doi: 10.3389/fped.2021.793400 (PMC8829565; doi:10.3389/fped.2021.793400)
Supplement: Supplementary file 1 [file Table_1.DOCX]

**Supplemental Table 1: Summary of literature search strategy**

| Database | Search terms |
| --- | --- |
| Pubmed  Science-direct  Web of Science | (“caesarean section” OR “cesarean section” OR “caesarean delivery” OR “cesarean delivery” OR “Planned cesarean” OR “planned caesarean” OR “elective caesarean” OR “elective cesarean”) AND ( “weight” OR “fat*” OR “adiposity” OR “obesity” OR “BMI” “physical growth” “physical development”) AND (“child*” OR “adolescen*” OR “infant” OR “toddler” OR “offspring”) |
| CNKI  Wanfang Database (in Chinese)  China Biology Medicine disc databases | Similar Chinese technical terms were adopted to search for eligible articles. |
